# 7-Benzyl­oxymethyl-9-bromo-6-chloro-9-deaza­purine

**DOI:** 10.1107/S1600536809050879

**Published:** 2009-12-16

**Authors:** Graeme J. Gainsford, Jennifer M. Mason, Shivali A. Gulab

**Affiliations:** a Industrial Research Limited, PO Box 31-310, Lower Hutt, New Zealand

## Abstract

The title compound, C_14_H_11_BrClN_3_O, crystallizes with two independent mol­ecules in the asymmetric unit. In the crystal, the molecules are linked by C—N⋯Br halogen bonds, as well as weak methyl­ene C—H⋯π, phenyl C—H⋯π, C—H⋯Br and phenyl C—H⋯O(ether) inter­actions.

## Related literature

For synthetic details, see Clinch *et al.* (2010 ▶). For Br⋯N halogen bonding, see: Kubicki (2004 ▶); Metrangolo *et al.* (2008 ▶). For a related structure, see: Sakore & Sobell (1969 ▶).
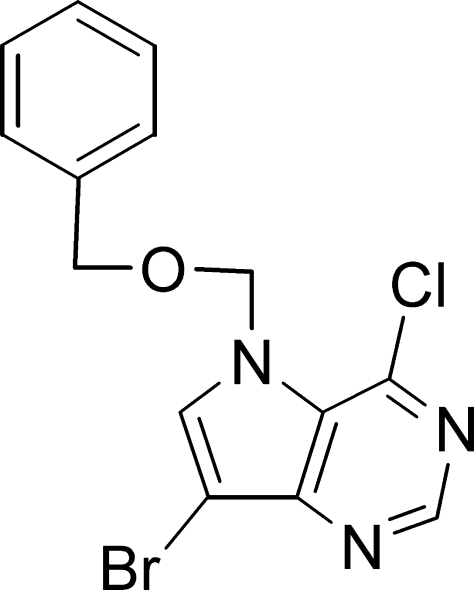


## Experimental

### 

#### Crystal data

C_14_H_11_BrClN_3_O
*M* *_r_* = 352.62
Triclinic, 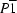

*a* = 7.8999 (2) Å
*b* = 11.5023 (4) Å
*c* = 15.5571 (5) Å
α = 86.111 (2)°
β = 84.564 (2)°
γ = 89.037 (2)°
*V* = 1403.95 (8) Å^3^
*Z* = 4
Mo *K*α radiation
μ = 3.12 mm^−1^
*T* = 118 K
0.41 × 0.21 × 0.02 mm

#### Data collection

Bruker APEXII CCD diffractometer
Absorption correction: multi-scan (Blessing, 1995 ▶) *T*_min_ = 0.606, *T*_max_ = 0.747
40101 measured reflections
9403 independent reflections
6435 reflections with *I* > 2σ(*I*)
*R*_int_ = 0.060

#### Refinement

*R*[*F* ^2^ > 2σ(*F* ^2^)] = 0.039
*wR*(*F* ^2^) = 0.081
*S* = 1.01
9403 reflections
361 parameters
H-atom parameters constrained
Δρ_max_ = 0.54 e Å^−3^
Δρ_min_ = −0.45 e Å^−3^

### 

Data collection: *APEX2* (Bruker, 2005 ▶); cell refinement: *SAINT* (Bruker, 2005 ▶); data reduction: *SAINT* and *SADABS* (Bruker, 2005 ▶); program(s) used to solve structure: *SHELXS97* (Sheldrick, 2008 ▶); program(s) used to refine structure: *SHELXL97* (Sheldrick, 2008 ▶); molecular graphics: *ORTEP-3* (Farrugia, 1997 ▶) and *PLATON* (Spek, 2009 ▶); software used to prepare material for publication: *SHELXL97* and *PLATON*.

## Supplementary Material

Crystal structure: contains datablocks global, I. DOI: 10.1107/S1600536809050879/wn2368sup1.cif

Structure factors: contains datablocks I. DOI: 10.1107/S1600536809050879/wn2368Isup2.hkl

Additional supplementary materials:  crystallographic information; 3D view; checkCIF report

## Figures and Tables

**Table 1:** Selected torsion angles (°)

C5—N7—C8—C9	0.3 (3)
N7—C10—O11—C12	−68.2 (3)
C10—O11—C12—C13	−172.6 (2)
C5′—N7′—C8′—C9′	0.1 (3)
N7′—C10′—O11′—C12′	−77.3 (2)
C10′—O11′—C12′—C13′	−161.5 (2)

**Table 2:** Hydrogen-bond geometry (Å, °)

*D*—H⋯*A*	*D*—H	H⋯*A*	*D*⋯*A*	*D*—H⋯*A*
C2—H2⋯Br9^i^	0.95	2.90	3.805 (3)	159
C8—H8⋯N1^ii^	0.95	2.46	3.394 (3)	166
C8′—H8′⋯N1′^ii^	0.95	2.48	3.410 (3)	166
C10′—H10*C*⋯Br9^iii^	0.99	2.78	3.700 (2)	156
C15—H15⋯O11′^iv^	0.95	2.60	3.497 (4)	158
C10—H10*A*⋯*Cg*1^v^	0.99	2.60	3.466 (3)	146
C14—H14⋯*Cg*2^iv^	0.95	2.87	3.794 (3)	165

Symmetry codes: (i) 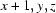; (ii) 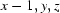; (iii) 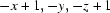; (iv) 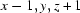; (v) 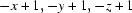. *Cg*1 and *Cg*2 are the centroids of the N7′,C4′,C5′,C8′,C9′ and C13′–C18′ rings, respectively.

**Table 3:** C—N⋯Br inter­actions (Å, °)

C—N⋯Br	C—N	N⋯Br	C⋯Br	C—N⋯Br
C2—N3⋯Br9′^vi^	1.320 (3)	2.964 (2)	3.457 (3)	100.45 (17)
C2′—N3′⋯Br^vii^	1.324 (3)	3.043 (2)	3.565 (3)	102.17 (14)

Symmetry codes: (vi) 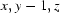; (vii) 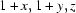.

**Fig. 1.**
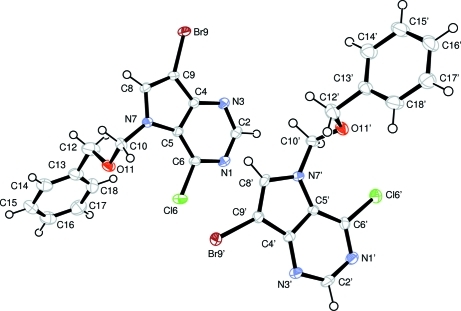
Asymmetric unit of the title compound (Farrugia, 1997).

**Fig. 2.**
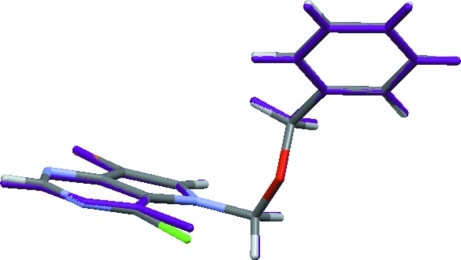
Overlap view of the two molecules (Mercury 2.3, Macrae et al. (2008)). Single coloured (purple) molecule is primed molecule 2.

**Fig. 3.**
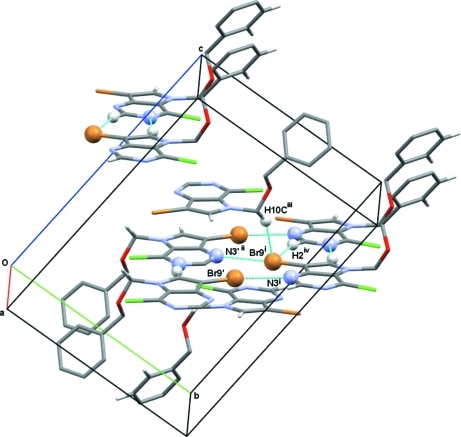
A packing view (Mercury 2.3, Macrae et al. (2008)) of the unit cell, highlighting the layers formed by Br···N bonding in the crystal structure (Table 3). Some of the other weak interactions (Table 2) are shown, with contact atoms in ball mode; only H atoms involved in short contacts are included. Symmetry codes: (i) x, 1 + y,z (ii) x - 1,y,z (iii) 1 - x,1 - y,1 - z (iv) x - 1,1 + y,z.

## supplementary crystallographic information

### Comment

The title compound was prepared for incorporation into potential transition state analogue inhibitors of human methylthioadenosine phosphorylase and bacterial methylthioadenosine/S-adenosylhomocysteine nucleosidase (Clinch *et al.*, 2010).

The asymmetric unit of the title compound contains two independent 7-benzyloxymethyl-9-bromo-6-chloro-9-deazapurine molecules (Fig. 1, Table 1) which are nearly identical. The molecular labels are related by the addition of a prime (*e.g.* molecule 1, Br9 and molecule 2, Br9'). The overlay figure (Fig. 2) illustrates the rmsd fit of 0.077 Å, with maximum deviation at the Cl6 (C16') atom sites of 0.274 Å. The five-membered rings (*i.e.* C4,C5,C8,C9,N7) are planar (maximum deviations 0.004 (2), 0.001 (2) Å), as are the fused six-membered rings (C4,C5,C6,N1,C2,N3) with maximum deviations 0.017 (2), 0.010 (2) Å; the angles between these two planes are 2.62 (13) and 1.00 (12)°, respectively. The dihedral angles between the mean planes through the deazapurine ring systems and the phenyl rings are 52.76 (12) and 58.02 (11)°.

Torsion angles (Table 1) are similar to those observed earlier in 9-ethyl-8-bromohypoxanthine (Sakore & Sobell, 1969).

Lattice binding is provided principally by Br···N halogen bonding (Metrangolo *et al.*, 2008; Kubicki, 2004) between the two independent molecules, forming layers parallel to the (0,1,2) plane (Table 3; Fig. 3). Inter-layer links are provided by weak methylene C–H···π, phenyl C–H···π, C–H···Br, and phenyl C–H···O(ether) interactions (Table 2). In Table 2, *Cg*1 and *Cg*2 are the centroids of N7',C4',C5',C8',C9' and C13'–C18', respectively.

### Experimental

Synthetic details are given in Clinch *et al.* (2010).

### Refinement

Data was restricted to 2θ = 64°. All carbon-bound H atoms were constrained to their expected geometries [C—H 0.95, 0.99 Å] and refined with *U*_iso_(H) = 1.2*U*_eq_(parent atom). All non-hydrogen atoms were refined with anisotropic thermal parameters.

### Crystal data

**Table:**

C_14_H_11_BrClN_3_O	*Z* = 4
*M**_r_* = 352.62	*F*(000) = 704
Triclinic, *P*1	*D*_x_ = 1.668 Mg m^−^^3^
Hall symbol: -P 1	Mo *K*α radiation, λ = 0.71073 Å
*a* = 7.8999 (2) Å	Cell parameters from 7237 reflections
*b* = 11.5023 (4) Å	θ = 2.3–28.2°
*c* = 15.5571 (5) Å	µ = 3.12 mm^−^^1^
α = 86.111 (2)°	*T* = 118 K
β = 84.564 (2)°	Plate, colourless
γ = 89.037 (2)°	0.41 × 0.21 × 0.02 mm
*V* = 1403.95 (8) Å^3^	

### Data collection

**Table:**

Bruker APEXII CCD diffractometer	9403 independent reflections
Radiation source: fine-focus sealed tube	6435 reflections with *I* > 2σ(*I*)
graphite	*R*_int_ = 0.060
Detector resolution: 8.333 pixels mm^-1^	θ_max_ = 32.0°, θ_min_ = 2.8°
φ and ω scans	*h* = −11→11
Absorption correction: multi-scan (Blessing, 1995)	*k* = −17→17
*T*_min_ = 0.606, *T*_max_ = 0.747	*l* = −23→23
40101 measured reflections	

### Refinement

**Table:**

Refinement on *F*^2^	Primary atom site location: structure-invariant direct methods
Least-squares matrix: full	Secondary atom site location: difference Fourier map
*R*[*F*^2^ > 2σ(*F*^2^)] = 0.039	Hydrogen site location: inferred from neighbouring sites
*wR*(*F*^2^) = 0.081	H-atom parameters constrained
*S* = 1.01	*w* = 1/[σ^2^(*F*_o_^2^) + (0.0269*P*)^2^ + 0.5143*P*] where *P* = (*F*_o_^2^ + 2*F*_c_^2^)/3
9403 reflections	(Δ/σ)_max_ = 0.002
361 parameters	Δρ_max_ = 0.54 e Å^−^^3^
0 restraints	Δρ_min_ = −0.44 e Å^−^^3^

### Special details

**Table:**

Geometry. All e.s.d.'s (except the e.s.d. in the dihedral angle between two l.s. planes) are estimated using the full covariance matrix. The cell e.s.d.'s are taken into account individually in the estimation of e.s.d.'s in distances, angles and torsion angles; correlations between e.s.d.'s in cell parameters are only used when they are defined by crystal symmetry. An approximate (isotropic) treatment of cell e.s.d.'s is used for estimating e.s.d.'s involving l.s. planes.
Refinement. Refinement of *F*^2^ against ALL reflections. The weighted *R*-factor *wR* andgoodness of fit *S* are based on *F*^2^, conventional *R*-factors *R* are basedon *F*, with *F* set to zero for negative *F*^2^. The threshold expression of*F*^2^ > σ(*F*^2^) is used only for calculating *R*-factors(gt) *etc*. and isnot relevant to the choice of reflections for refinement. *R*-factors basedon *F*^2^ are statistically about twice as large as those based on *F*, and *R*-factors based on ALL data will be even larger.

### Fractional atomic coordinates and isotropic or equivalent isotropic displacement parameters (Å^2^)

**Table:**

	*x*	*y*	*z*	*U*_iso_*/*U*_eq_	
Br9	0.07829 (3)	−0.13291 (2)	0.558649 (16)	0.02240 (6)	
Cl6	0.55993 (8)	0.24034 (6)	0.75030 (4)	0.03239 (15)	
N1	0.6523 (2)	0.0585 (2)	0.66667 (15)	0.0294 (5)	
C2	0.6243 (3)	−0.0336 (3)	0.62176 (19)	0.0337 (7)	
H2	0.7222	−0.0760	0.6010	0.040*	
N3	0.4768 (2)	−0.07252 (19)	0.60296 (14)	0.0271 (5)	
C4	0.3419 (3)	−0.0102 (2)	0.63424 (15)	0.0204 (5)	
C5	0.3551 (3)	0.0859 (2)	0.68449 (15)	0.0194 (5)	
C6	0.5190 (3)	0.1184 (2)	0.69711 (16)	0.0226 (5)	
N7	0.1938 (2)	0.12957 (17)	0.70536 (13)	0.0200 (4)	
C8	0.0815 (3)	0.0629 (2)	0.66795 (15)	0.0207 (5)	
H8	−0.0383	0.0745	0.6720	0.025*	
C9	0.1670 (3)	−0.0223 (2)	0.62432 (15)	0.0187 (5)	
C10	0.1459 (3)	0.2263 (2)	0.75875 (16)	0.0244 (5)	
H10A	0.2034	0.2975	0.7321	0.029*	
H10B	0.0218	0.2400	0.7592	0.029*	
O11	0.1867 (2)	0.20846 (15)	0.84366 (11)	0.0265 (4)	
C12	0.0892 (4)	0.1195 (3)	0.89288 (19)	0.0465 (8)	
H12A	0.1187	0.0426	0.8702	0.056*	
H12B	−0.0336	0.1344	0.8888	0.056*	
C13	0.1286 (4)	0.1199 (3)	0.98518 (18)	0.0343 (6)	
C14	0.0310 (4)	0.1865 (2)	1.0417 (2)	0.0362 (7)	
H14	−0.0592	0.2333	1.0215	0.043*	
C15	0.0629 (4)	0.1861 (3)	1.12723 (19)	0.0377 (7)	
H15	−0.0061	0.2318	1.1656	0.045*	
C16	0.1938 (4)	0.1200 (3)	1.1573 (2)	0.0395 (7)	
H16	0.2158	0.1200	1.2163	0.047*	
C17	0.2929 (4)	0.0539 (3)	1.1013 (2)	0.0446 (8)	
H17	0.3841	0.0082	1.1215	0.054*	
C18	0.2602 (4)	0.0536 (3)	1.0156 (2)	0.0410 (7)	
H18	0.3289	0.0073	0.9773	0.049*	
Br9'	0.59248 (3)	0.72770 (2)	0.500913 (16)	0.02152 (6)	
Cl6'	1.15241 (7)	0.36355 (6)	0.28870 (4)	0.02845 (14)	
N1'	1.2108 (2)	0.54047 (18)	0.37585 (13)	0.0228 (4)	
C2'	1.1644 (3)	0.6305 (2)	0.42386 (17)	0.0257 (5)	
H2'	1.2547	0.6744	0.4411	0.031*	
N3'	1.0089 (2)	0.66584 (18)	0.45013 (13)	0.0223 (4)	
C4'	0.8857 (3)	0.6018 (2)	0.42261 (15)	0.0182 (5)	
C5'	0.9182 (3)	0.5069 (2)	0.37055 (14)	0.0175 (4)	
C6'	1.0881 (3)	0.4791 (2)	0.34985 (15)	0.0197 (5)	
N7'	0.7642 (2)	0.46355 (17)	0.35311 (12)	0.0190 (4)	
C8'	0.6378 (3)	0.5300 (2)	0.39368 (15)	0.0199 (5)	
H8'	0.5194	0.5186	0.3921	0.024*	
C9'	0.7066 (3)	0.6140 (2)	0.43611 (15)	0.0190 (5)	
C10'	0.7317 (3)	0.3645 (2)	0.30284 (16)	0.0225 (5)	
H10C	0.8014	0.2971	0.3222	0.027*	
H10D	0.6107	0.3427	0.3150	0.027*	
O11'	0.76778 (19)	0.38677 (15)	0.21388 (11)	0.0250 (4)	
C12'	0.6421 (3)	0.4559 (3)	0.17201 (17)	0.0320 (6)	
H12C	0.6546	0.5392	0.1820	0.038*	
H12D	0.5264	0.4312	0.1953	0.038*	
C13'	0.6698 (3)	0.4377 (2)	0.07720 (17)	0.0271 (6)	
C14'	0.5969 (3)	0.3438 (3)	0.04404 (19)	0.0361 (7)	
H14'	0.5247	0.2928	0.0810	0.043*	
C15'	0.6289 (4)	0.3240 (3)	−0.04277 (19)	0.0408 (7)	
H15'	0.5790	0.2592	−0.0652	0.049*	
C16'	0.7331 (3)	0.3979 (3)	−0.09698 (19)	0.0371 (7)	
H16'	0.7555	0.3842	−0.1565	0.045*	
C17'	0.8045 (4)	0.4920 (3)	−0.06387 (19)	0.0397 (7)	
H17'	0.8758	0.5434	−0.1008	0.048*	
C18'	0.7726 (3)	0.5115 (3)	0.02262 (18)	0.0357 (7)	
H18'	0.8221	0.5766	0.0448	0.043*	

### Atomic displacement parameters (Å^2^)

**Table:**

	*U*^11^	*U*^22^	*U*^33^	*U*^12^	*U*^13^	*U*^23^
Br9	0.01902 (11)	0.02210 (13)	0.02763 (14)	−0.00162 (8)	−0.00850 (9)	−0.00370 (10)
Cl6	0.0302 (3)	0.0355 (4)	0.0336 (4)	−0.0109 (3)	−0.0062 (3)	−0.0109 (3)
N1	0.0160 (9)	0.0408 (14)	0.0332 (13)	−0.0029 (8)	−0.0051 (8)	−0.0111 (11)
C2	0.0144 (11)	0.0463 (18)	0.0423 (18)	0.0040 (10)	−0.0035 (10)	−0.0164 (14)
N3	0.0163 (9)	0.0343 (13)	0.0323 (13)	0.0021 (8)	−0.0042 (8)	−0.0107 (10)
C4	0.0173 (10)	0.0242 (13)	0.0198 (13)	−0.0013 (8)	−0.0034 (8)	0.0004 (10)
C5	0.0167 (10)	0.0251 (13)	0.0166 (12)	−0.0010 (8)	−0.0030 (8)	−0.0005 (10)
C6	0.0216 (11)	0.0289 (14)	0.0182 (12)	−0.0054 (9)	−0.0058 (9)	−0.0025 (10)
N7	0.0170 (9)	0.0223 (11)	0.0213 (11)	0.0000 (7)	−0.0034 (7)	−0.0049 (8)
C8	0.0149 (10)	0.0263 (13)	0.0214 (13)	−0.0008 (8)	−0.0044 (8)	−0.0019 (10)
C9	0.0174 (10)	0.0204 (12)	0.0191 (12)	−0.0024 (8)	−0.0052 (8)	−0.0022 (9)
C10	0.0272 (12)	0.0232 (13)	0.0230 (14)	0.0031 (9)	−0.0011 (9)	−0.0045 (11)
O11	0.0326 (9)	0.0286 (10)	0.0185 (9)	−0.0086 (7)	−0.0009 (7)	−0.0031 (8)
C12	0.062 (2)	0.051 (2)	0.0268 (17)	−0.0310 (16)	−0.0012 (14)	0.0029 (14)
C13	0.0421 (15)	0.0349 (16)	0.0258 (15)	−0.0169 (12)	−0.0013 (11)	0.0004 (12)
C14	0.0421 (16)	0.0290 (16)	0.0390 (18)	−0.0022 (12)	−0.0110 (13)	−0.0023 (13)
C15	0.0475 (17)	0.0343 (17)	0.0324 (17)	−0.0003 (13)	−0.0042 (13)	−0.0100 (13)
C16	0.0494 (17)	0.0420 (18)	0.0281 (16)	−0.0092 (14)	−0.0097 (13)	0.0007 (14)
C17	0.0379 (16)	0.049 (2)	0.047 (2)	−0.0003 (14)	−0.0113 (14)	0.0032 (16)
C18	0.0364 (15)	0.0460 (19)	0.0393 (19)	−0.0044 (13)	0.0072 (13)	−0.0086 (15)
Br9'	0.01786 (10)	0.02498 (14)	0.02185 (13)	0.00062 (8)	−0.00097 (8)	−0.00401 (10)
Cl6'	0.0234 (3)	0.0316 (3)	0.0305 (4)	0.0022 (2)	0.0014 (2)	−0.0094 (3)
N1'	0.0177 (9)	0.0271 (12)	0.0235 (12)	−0.0024 (8)	−0.0023 (7)	−0.0005 (9)
C2'	0.0148 (10)	0.0315 (14)	0.0315 (15)	−0.0057 (9)	−0.0052 (9)	−0.0012 (11)
N3'	0.0187 (9)	0.0254 (11)	0.0235 (11)	−0.0034 (8)	−0.0044 (8)	−0.0029 (9)
C4'	0.0177 (10)	0.0192 (12)	0.0178 (12)	−0.0013 (8)	−0.0034 (8)	0.0009 (9)
C5'	0.0166 (9)	0.0222 (12)	0.0142 (11)	−0.0025 (8)	−0.0035 (8)	−0.0017 (9)
C6'	0.0199 (10)	0.0236 (12)	0.0151 (12)	0.0011 (9)	0.0007 (8)	0.0001 (10)
N7'	0.0151 (8)	0.0243 (11)	0.0187 (11)	−0.0031 (7)	−0.0040 (7)	−0.0045 (8)
C8'	0.0122 (9)	0.0282 (13)	0.0189 (12)	−0.0001 (8)	−0.0013 (8)	0.0021 (10)
C9'	0.0188 (10)	0.0224 (12)	0.0158 (12)	−0.0008 (8)	−0.0014 (8)	−0.0007 (9)
C10'	0.0236 (11)	0.0229 (13)	0.0219 (13)	−0.0037 (9)	−0.0056 (9)	−0.0037 (10)
O11'	0.0226 (8)	0.0349 (10)	0.0187 (9)	0.0060 (7)	−0.0058 (6)	−0.0064 (8)
C12'	0.0312 (13)	0.0412 (17)	0.0249 (15)	0.0107 (11)	−0.0078 (10)	−0.0060 (12)
C13'	0.0260 (12)	0.0348 (15)	0.0219 (14)	0.0063 (10)	−0.0079 (10)	−0.0055 (11)
C14'	0.0411 (15)	0.0394 (17)	0.0282 (16)	−0.0068 (12)	−0.0063 (12)	0.0005 (13)
C15'	0.0510 (17)	0.0438 (19)	0.0299 (17)	−0.0083 (14)	−0.0098 (13)	−0.0089 (14)
C16'	0.0405 (15)	0.0497 (19)	0.0226 (15)	0.0026 (13)	−0.0079 (11)	−0.0064 (13)
C17'	0.0404 (16)	0.052 (2)	0.0272 (16)	−0.0108 (13)	−0.0059 (12)	0.0008 (14)
C18'	0.0412 (15)	0.0386 (17)	0.0294 (16)	−0.0075 (12)	−0.0114 (12)	−0.0052 (13)

### Geometric parameters (Å, °)

**Table:**

Br9—C9	1.870 (2)	Br9'—C9'	1.873 (2)
Cl6—C6	1.726 (2)	Cl6'—C6'	1.728 (2)
N1—C6	1.316 (3)	N1'—C6'	1.318 (3)
N1—C2	1.341 (3)	N1'—C2'	1.344 (3)
C2—N3	1.320 (3)	C2'—N3'	1.324 (3)
C2—H2	0.9500	C2'—H2'	0.9500
N3—C4	1.345 (3)	N3'—C4'	1.348 (3)
C4—C5	1.407 (3)	C4'—C5'	1.409 (3)
C4—C9	1.415 (3)	C4'—C9'	1.417 (3)
C5—N7	1.380 (3)	C5'—N7'	1.379 (3)
C5—C6	1.390 (3)	C5'—C6'	1.388 (3)
N7—C8	1.377 (3)	N7'—C8'	1.379 (3)
N7—C10	1.457 (3)	N7'—C10'	1.465 (3)
C8—C9	1.363 (3)	C8'—C9'	1.355 (3)
C8—H8	0.9500	C8'—H8'	0.9500
C10—O11	1.390 (3)	C10'—O11'	1.394 (3)
C10—H10A	0.9900	C10'—H10C	0.9900
C10—H10B	0.9900	C10'—H10D	0.9900
O11—C12	1.427 (3)	O11'—C12'	1.438 (3)
C12—C13	1.498 (4)	C12'—C13'	1.499 (4)
C12—H12A	0.9900	C12'—H12C	0.9900
C12—H12B	0.9900	C12'—H12D	0.9900
C13—C14	1.378 (4)	C13'—C18'	1.377 (4)
C13—C18	1.380 (4)	C13'—C14'	1.385 (4)
C14—C15	1.377 (4)	C14'—C15'	1.384 (4)
C14—H14	0.9500	C14'—H14'	0.9500
C15—C16	1.372 (4)	C15'—C16'	1.381 (4)
C15—H15	0.9500	C15'—H15'	0.9500
C16—C17	1.376 (4)	C16'—C17'	1.380 (4)
C16—H16	0.9500	C16'—H16'	0.9500
C17—C18	1.382 (4)	C17'—C18'	1.378 (4)
C17—H17	0.9500	C17'—H17'	0.9500
C18—H18	0.9500	C18'—H18'	0.9500
			
C6—N1—C2	117.6 (2)	C6'—N1'—C2'	117.19 (19)
N3—C2—N1	127.8 (2)	N3'—C2'—N1'	128.2 (2)
N3—C2—H2	116.1	N3'—C2'—H2'	115.9
N1—C2—H2	116.1	N1'—C2'—H2'	115.9
C2—N3—C4	113.9 (2)	C2'—N3'—C4'	113.4 (2)
N3—C4—C5	123.5 (2)	N3'—C4'—C5'	123.7 (2)
N3—C4—C9	130.0 (2)	N3'—C4'—C9'	129.7 (2)
C5—C4—C9	106.56 (19)	C5'—C4'—C9'	106.58 (19)
N7—C5—C6	135.4 (2)	N7'—C5'—C6'	135.8 (2)
N7—C5—C4	108.42 (18)	N7'—C5'—C4'	108.19 (18)
C6—C5—C4	116.1 (2)	C6'—C5'—C4'	116.05 (19)
N1—C6—C5	121.1 (2)	N1'—C6'—C5'	121.4 (2)
N1—C6—Cl6	116.47 (17)	N1'—C6'—Cl6'	115.92 (17)
C5—C6—Cl6	122.42 (19)	C5'—C6'—Cl6'	122.65 (18)
C8—N7—C5	107.50 (19)	C8'—N7'—C5'	107.52 (19)
C8—N7—C10	124.96 (19)	C8'—N7'—C10'	123.80 (18)
C5—N7—C10	127.53 (19)	C5'—N7'—C10'	128.67 (19)
C9—C8—N7	110.16 (19)	C9'—C8'—N7'	110.33 (19)
C9—C8—H8	124.9	C9'—C8'—H8'	124.8
N7—C8—H8	124.9	N7'—C8'—H8'	124.8
C8—C9—C4	107.4 (2)	C8'—C9'—C4'	107.4 (2)
C8—C9—Br9	128.03 (17)	C8'—C9'—Br9'	127.83 (17)
C4—C9—Br9	124.56 (18)	C4'—C9'—Br9'	124.78 (17)
O11—C10—N7	113.9 (2)	O11'—C10'—N7'	113.58 (19)
O11—C10—H10A	108.8	O11'—C10'—H10C	108.9
N7—C10—H10A	108.8	N7'—C10'—H10C	108.9
O11—C10—H10B	108.8	O11'—C10'—H10D	108.9
N7—C10—H10B	108.8	N7'—C10'—H10D	108.9
H10A—C10—H10B	107.7	H10C—C10'—H10D	107.7
C10—O11—C12	113.6 (2)	C10'—O11'—C12'	115.05 (18)
O11—C12—C13	108.2 (2)	O11'—C12'—C13'	107.2 (2)
O11—C12—H12A	110.1	O11'—C12'—H12C	110.3
C13—C12—H12A	110.1	C13'—C12'—H12C	110.3
O11—C12—H12B	110.1	O11'—C12'—H12D	110.3
C13—C12—H12B	110.1	C13'—C12'—H12D	110.3
H12A—C12—H12B	108.4	H12C—C12'—H12D	108.5
C14—C13—C18	118.8 (3)	C18'—C13'—C14'	119.2 (3)
C14—C13—C12	119.8 (3)	C18'—C13'—C12'	120.5 (2)
C18—C13—C12	121.4 (3)	C14'—C13'—C12'	120.2 (3)
C15—C14—C13	120.7 (3)	C15'—C14'—C13'	120.2 (3)
C15—C14—H14	119.7	C15'—C14'—H14'	119.9
C13—C14—H14	119.7	C13'—C14'—H14'	119.9
C16—C15—C14	120.4 (3)	C16'—C15'—C14'	120.3 (3)
C16—C15—H15	119.8	C16'—C15'—H15'	119.9
C14—C15—H15	119.8	C14'—C15'—H15'	119.9
C15—C16—C17	119.3 (3)	C17'—C16'—C15'	119.4 (3)
C15—C16—H16	120.3	C17'—C16'—H16'	120.3
C17—C16—H16	120.3	C15'—C16'—H16'	120.3
C16—C17—C18	120.3 (3)	C18'—C17'—C16'	120.2 (3)
C16—C17—H17	119.9	C18'—C17'—H17'	119.9
C18—C17—H17	119.9	C16'—C17'—H17'	119.9
C13—C18—C17	120.5 (3)	C13'—C18'—C17'	120.7 (3)
C13—C18—H18	119.8	C13'—C18'—H18'	119.7
C17—C18—H18	119.8	C17'—C18'—H18'	119.7
			
C6—N1—C2—N3	0.7 (5)	C6'—N1'—C2'—N3'	1.0 (4)
N1—C2—N3—C4	−0.4 (4)	N1'—C2'—N3'—C4'	−1.0 (4)
C2—N3—C4—C5	−1.7 (4)	C2'—N3'—C4'—C5'	−0.4 (3)
C2—N3—C4—C9	177.1 (3)	C2'—N3'—C4'—C9'	−178.8 (2)
N3—C4—C5—N7	179.8 (2)	N3'—C4'—C5'—N7'	−178.6 (2)
C9—C4—C5—N7	0.8 (3)	C9'—C4'—C5'—N7'	0.1 (3)
N3—C4—C5—C6	3.3 (4)	N3'—C4'—C5'—C6'	1.7 (3)
C9—C4—C5—C6	−175.7 (2)	C9'—C4'—C5'—C6'	−179.6 (2)
C2—N1—C6—C5	1.2 (4)	C2'—N1'—C6'—C5'	0.5 (3)
C2—N1—C6—Cl6	−177.1 (2)	C2'—N1'—C6'—Cl6'	−179.70 (19)
N7—C5—C6—N1	−178.2 (3)	N7'—C5'—C6'—N1'	178.7 (2)
C4—C5—C6—N1	−3.0 (4)	C4'—C5'—C6'—N1'	−1.7 (3)
N7—C5—C6—Cl6	0.0 (4)	N7'—C5'—C6'—Cl6'	−1.0 (4)
C4—C5—C6—Cl6	175.20 (18)	C4'—C5'—C6'—Cl6'	178.51 (17)
C6—C5—N7—C8	174.8 (3)	C6'—C5'—N7'—C8'	179.5 (3)
C4—C5—N7—C8	−0.7 (3)	C4'—C5'—N7'—C8'	−0.1 (3)
C6—C5—N7—C10	−6.7 (4)	C6'—C5'—N7'—C10'	0.6 (4)
C4—C5—N7—C10	177.9 (2)	C4'—C5'—N7'—C10'	−179.0 (2)
C5—N7—C8—C9	0.3 (3)	C5'—N7'—C8'—C9'	0.1 (3)
C10—N7—C8—C9	−178.3 (2)	C10'—N7'—C8'—C9'	179.0 (2)
N7—C8—C9—C4	0.2 (3)	N7'—C8'—C9'—C4'	0.0 (3)
N7—C8—C9—Br9	−177.46 (17)	N7'—C8'—C9'—Br9'	179.70 (17)
N3—C4—C9—C8	−179.5 (3)	N3'—C4'—C9'—C8'	178.6 (2)
C5—C4—C9—C8	−0.6 (3)	C5'—C4'—C9'—C8'	−0.1 (3)
N3—C4—C9—Br9	−1.8 (4)	N3'—C4'—C9'—Br9'	−1.1 (4)
C5—C4—C9—Br9	177.16 (17)	C5'—C4'—C9'—Br9'	−179.77 (17)
C8—N7—C10—O11	116.7 (2)	C8'—N7'—C10'—O11'	107.8 (2)
C5—N7—C10—O11	−61.7 (3)	C5'—N7'—C10'—O11'	−73.4 (3)
N7—C10—O11—C12	−68.2 (3)	N7'—C10'—O11'—C12'	−77.3 (2)
C10—O11—C12—C13	−172.6 (2)	C10'—O11'—C12'—C13'	−161.5 (2)
O11—C12—C13—C14	91.4 (3)	O11'—C12'—C13'—C18'	−92.3 (3)
O11—C12—C13—C18	−89.4 (3)	O11'—C12'—C13'—C14'	85.3 (3)
C18—C13—C14—C15	−0.7 (4)	C18'—C13'—C14'—C15'	0.7 (4)
C12—C13—C14—C15	178.6 (3)	C12'—C13'—C14'—C15'	−177.0 (3)
C13—C14—C15—C16	0.7 (4)	C13'—C14'—C15'—C16'	−0.3 (5)
C14—C15—C16—C17	−0.2 (5)	C14'—C15'—C16'—C17'	−0.2 (5)
C15—C16—C17—C18	−0.3 (5)	C15'—C16'—C17'—C18'	0.3 (4)
C14—C13—C18—C17	0.2 (4)	C14'—C13'—C18'—C17'	−0.6 (4)
C12—C13—C18—C17	−179.0 (3)	C12'—C13'—C18'—C17'	177.0 (3)
C16—C17—C18—C13	0.3 (5)	C16'—C17'—C18'—C13'	0.1 (5)

### Hydrogen-bond geometry (Å, °)

**Table:**

*D*—H···*A*	*D*—H	H···*A*	*D*···*A*	*D*—H···*A*
C2—H2···Br9^i^	0.95	2.90	3.805 (3)	159
C8—H8···N1^ii^	0.95	2.46	3.394 (3)	166
C8'—H8'···N1'^ii^	0.95	2.48	3.410 (3)	166
C10'—H10C···Br9^iii^	0.99	2.78	3.700 (2)	156
C15—H15···O11'^iv^	0.95	2.60	3.497 (4)	158
C10—H10A···Cg1^v^	0.99	2.60	3.466 (3)	146
C14—H14···Cg2^iv^	0.95	2.87	3.794 (3)	165

Symmetry codes: (i) *x*+1, *y*, *z*; (ii) *x*−1, *y*, *z*; (iii) −*x*+1, −*y*, −*z*+1; (iv) *x*−1, *y*, *z*+1; (v) −*x*+1, −*y*+1, −*z*+1.

### Table 3 C—N···Br interactions (Å, °)

**Table:**

C—N···Br	C—N	N···Br	C···Br	C—N···Br
C2—N3···Br9'^vi^	1.320 (3)	2.964 (2)	3.457 (3)	100.45 (17)
C2'—N3'···Br^vii^	1.324 (3)	3.043 (2)	3.565 (3)	102.17 (14)

Symmetry codes: (vi) x, y-1, z; (vii) 1+x, 1+y, z.
